# Environmental structure impacts microbial composition and secondary metabolism

**DOI:** 10.1038/s43705-022-00097-5

**Published:** 2022-02-03

**Authors:** Emily N. Junkins, Joseph B. McWhirter, Laura-Isobel McCall, Bradley S. Stevenson

**Affiliations:** 1 Department of Microbiology and Plant Biology, University of Oklahoma, Norman, OK USA; 2 Department of Chemistry and Biochemistry, University of Oklahoma, Norman, OK USA; 3 Department of Earth and Planetary Science, Northwestern University, Chicago, IL USA

**Keywords:** Microbial ecology, Metabolomics, Microbial ecology

## Abstract

Determining the drivers of microbial community assembly is a central theme of microbial ecology, and chemical ecologists seek to characterize how secondary metabolites mediate these assembly patterns. Environmental structure affects how communities assemble and what metabolic pathways aid in that assembly. Here, we bridged these two perspectives by addressing the chemical drivers of community assembly within a spatially structured landscape with varying oxygen availability. We hypothesized that structured environments would favor higher microbial diversity and metabolite diversity. We anticipated that the production of a compound would be more advantageous in a structured environment (less mixing) compared to an unstructured environment (more mixing), where the molecule would have a diminished local effect. We observed this to be partially true in our experiments: structured environments had similar microbial diversity compared to unstructured environments but differed significantly in the metabolites produced. We also found that structured environments selected for communities with higher evenness, rather than communities with higher richness. This supports the idea that when characterizing the drivers of community assembly, it matters less about *who is there* and more about *what they are doing*. Overall, these data contribute to a growing effort to approach microbial community assembly with interdisciplinary tools and perspectives.

## Introduction

A central focus of microbial ecology is characterizing the forces that govern microbial community assembly. Because microbial communities exist in spatial patches [1, 2] and assemble into cell aggregates [3, 4], interactions will play a major role in community assembly as populations establish complex networks that define how microbial communities function. For example, microbial interactions can drive geochemical processes [5–7] or nutrient uptake and host physiology in gut systems [8, 9]. Many microbial interactions are mediated by the production of secondary (specialized) metabolites that aid in communication [10] or competition [11]. Characterizing the chemical communication network in which microorganisms exist is crucial to understanding the rules that govern interactions at the population level [12, 13]. Where microbial ecology often halts is in bridging the gap between biodiversity/community assembly and chemical ecology/chemodiversity [14]. Because ecology is mediated by chemical agents at various temporal and spatial scales, the chemistry of a system is an important factor when characterizing a community’s function and stability [14, 15]. Since many microorganisms naturally exist in a spatially structured, heterogeneous environment like biofilms [3], it is important to characterize the effect of environmental structure on microbial community composition and function.

A structured environment contains many habitats defined by barriers, chemical or physical, which can then influence the structure of a microbial community and its spatiotemporal dynamics (i.e., habitat heterogeneity, niche construction, stability) [16–20]. Structured environments would lead to more environmental gradients and unstructured environments would harbor fewer environmental gradients (i.e., habitats) [21–24]. Structure can result abiotically from microscale gradients in nutrients or chemicals, like oxygen or light, as well as physical barriers, like surfaces [23, 25]. Structure can be established biotically through the metabolic activity of microorganisms themselves via nutrient degradation and the production of metabolites involved in signaling or competition [26–28]. Overall, gradients create spatial heterogeneity which affords more niches into which diverse groups can sort. It is as populations seek to assemble or compete in these gradients that microorganisms interact with each other [12] and where biodiversity and chemodiversity are intrinsically linked.

Structured environments provide a mosaic of habitats in which neighbors can affect each other and their surroundings [29]. The effect that structured environments have on clonal microbial populations has been characterized for some organisms, such as colicin-producing and colicin-sensitive *Escherichia coli* strains, where the structure of the environment and the population size determined if a producer strain could outcompete the sensitive strain [30]. A structured environment also selects for phenotype diversification in *Pseudomonas fluorescens* compared to a constantly mixed, unstructured environment [31]. In the case of *P. fluorescens*, structure provided vacant niches which allowed for different genotypes to partition along a gradient, particularly an oxygen gradient [31]. Another example using a mixture of algal species showed that spatial structure afforded by heterogenous stream flow rates selected for coexistence (diversity) where a uniform flow rate (unstructured) led to competitive exclusion [32]. Overall, structured environments can afford multiple niche spaces, selecting for both microbial and functional diversity [33], and thereby creating ecological opportunity [34]. It follows that where there is opportunity that can be exploited by multiple groups, there will be competition for resources as organisms seek to establish themselves. Unlike the previous examples, we sought to characterize community assembly and interactions in a structured environment using a complex microbial community.

We hypothesized that structured environments would select for the production of antimicrobial metabolites as a competitive advantage during community assembly. We anticipated that the production of a compound would be more advantageous in a structured environment (limited mixing), compared to an unstructured environment (more mixing) where the molecule would have a diminished local effect [35]. Specifically, a structured environment would keep the producer and compounds within close range so that a producer can incur the benefit from costly production [12, 30, 36]. Therefore, spatial structure will determine the effect molecules can have on a population. We also hypothesized that the static communities would be more diverse (i.e., higher richness) since differential gradients could allow for slow-growers to partition, compared to the shaking communities that had a highly oxygenated environment that could select for a few, fast-growers. To test these hypotheses, we took a diverse wastewater community, diluted it to obtain inocula of varying cell densities, and incubated them in static conditions to promote structure (e.g., oxygen gradients) or shaking conditions to disrupt any gradients [31]. Microbial communities associated with wastewater treatment facilities provide a diverse, and metabolically active community, in which many populations are interacting [37–39]. We measured the microbial community diversity over time via 16S rRNA gene amplicon sequencing and the metabolite diversity through untargeted metabolomics. We found that structured environments selected for different microbial communities and different chemical profiles.

## Materials and methods

### Sampling and experimental setup

Wastewater was collected from the aerator basin at the Norman Water Reclamation Facility, Norman, OK on 11 February 2020. The sample (50 mL volume) was homogenized vigorously on a vortex mixer for ~2 min to break up flocks and produce a slurry. The sample was serially diluted (1:10) in sterile R2B liquid medium to generate 5 different dilutions (dilution factors of 1 to 10,000) to be used for the starting inocula [40]. R2B medium was chosen as a low-nutrient medium designed to increase cultivable isolates from water treatment facilities [40]. Each dilution was inoculated into sterile R2B medium for a final volume of 35 mL. A total of 150 enrichments were made so that three replicates could be sampled destructively, representing each dilution (1e−00, 1e−01, 1e−02, 1e−03, 1e−04) for each sampling day (Day 0, 3, 6, 9, 12) and for each condition (shaking or static). A total of 15 uninoculated medium controls were made to be used at each time point (*n* = 3). All cultures were incubated at 30 °C, either shaking at 250 rpm or static (Supplementary Fig. 1).

For each time point, triplicate cultures from each inoculum size and condition were vortexed and pooled. From this pool, three 1.0 mL aliquots were used to generate a cell pellet for DNA extraction (see Supplementary Detailed Methods). Cells were resuspended in 750 µL Zymo bashing bead buffer (per Zymo Quick-DNA manufacturer protocol), transferred to Zymo bashing bead tubes (Zymo Research Corp., Irvine, CA, USA), homogenized for 45 seconds using a hand-held reciprocating saw with custom attachment (RYOBI, Anderson, SC, USA), and stored at −20 °C until DNA extraction. Lastly, 103 mL of ethyl acetate was added to the remaining volume of the pooled samples (~103 mL) and mixed to generate a 1:1 ratio of culture and solvent for an overnight, organic extraction at room temperature. Medium controls for each time point, described above, were pooled, sampled for DNA extraction, and prepared for ethyl acetate organic extraction as described above for the cultures to be used as blanks for both sequencing and metabolomic data.

### DNA extraction and sequencing

Before DNA extraction, each sample was thawed on ice and homogenized for 45 s using a hand-held reciprocating saw. DNA was extracted according to manufacturer specifications using the Zymo *Quick*-DNA Fungal/Bacterial Miniprep kit (Zymo Research Corp., Irvine, CA, USA). Samples were stored at −20 °C until extractions could be processed at once. For community characterization, a conserved region of the SSU rRNA gene of most bacteria, archaea, and eukarya was amplified using primers 515F-Y and 926R [41] following a previously published protocol [42]. The amplified fragments were purified using Sera-Mag magnetic beads (ThermoFisher, Waltham, MA, USA) with the AmPureXP (Beckman Coulter, Indianapolis, IN, USA) protocol at a final concentration of 1.8× v/v. After purification, each PCR product was used in a separate barcoding PCR (6 cycles) in 50 µL reactions to attach a unique barcode to amplicons of each library [42]. The now barcoded amplicons were purified using Sera-Mag (ThermoFisher, Waltham, MA, USA) beads with the AmPureXP (Beckman Coulter, Indianapolis, IN, USA) protocol to a final volume of 40 µL, quantified using the QuBit HS DS DNA assay kit (ThermoFisher, Waltham, MA, USA), and pooled in equimolar amounts. The pooled, barcoded amplicon libraries were then concentrated to a final volume of 100 µL (16.6 ng/µL) with an Amicon-Ultra filter (Millipore, Burlington, MA, USA) following manufacturer’s protocol. The combined amplicon libraries were denatured according to MiSeq library preparations protocol (Illumina, San Diego, CA, USA). The sample was loaded at a concentration of 10 pM and sequenced using 2 × 250 paired-end strategy on the MiSeq (Illumina San Diego, CA, USA) platform for 251 cycles.

### ASV detection

Barcodes from raw SSU rRNA amplicon sequences were trimmed and sequences were dereplicated using cutadapt v3.0 [43]. Demultiplexed reads were quality filtered, forward and reverse reads were merged (~372 bp) and amplicon sequence variants (ASVs) were inferred as a part of the *dada2* pipeline [44]. Taxonomy was assigned using the SILVA database v32 [45, 46]. Contaminants were removed via the r package *decontam* v. 1.10 using the frequency- and prevalence-based filtering with a threshold of 0.5 [47]. The final, experimental dataset consisted of ~1.4 million reads resulting in 1923 ASVs with a median of 9054 reads per samples.

### Tree building and community analysis

Sequences were aligned using the r package *DECIPHER* v2.0 [48, 49] and the r package *phangorn* v2.5.5 [50] was used to first construct a neighbor-joining tree to use as the starting point for a GTR + G + I maximum likelihood tree. Community analysis was done using the r package *phyloseq* v1.26.1 [51]. Richness was calculated using the r package *breakaway* which calculates error in richness estimation [52]. Evenness was calculated using Pielou’s index [53]. To test whether environmental structure influenced the richness and evenness of the microbial communities for each dilution, a Levene’s test was used to confirm homogeneity of variance [54] using the r package *car* [55] before a two-way ANOVA coupled with a Tukey’s honest significant difference test was used to compare differences between shaking and static cultures for each time point in PRISM v9 (GraphPad Software, San Diego, CA, USA). Dilutions were treated as independent communities and alpha diversity significance tests were run on each dilution separately.

To characterize beta diversity, distances were calculated using weighted UniFrac distances [56] and ordinated using Principal Coordinate Analysis (PCoA) calculated with 95% confidence ellipses. Statistical comparisons across cultivation conditions, dilution, and day were done using PERMANOVA with the adonis function in the *vegan* r package (~Dilution*Condition*Day) [57]. To understand the significance described in the PERMANOVA, a PERMDISP with the betadisper function in *vegan* was used to describe any within sample variance (i.e., across replicates) that could explain any significant differences detected in the PERMANOVA. Hypothesis testing via ANOVA with permutations (*n* = 999) was used with PERMDISP to determine any significant differences in variation within samples. To investigate changes in abundance of certain taxa between shaking and static conditions, the r package *corncob* was used to calculate differential abundances using a beta binomial model at the genus level [58]. This model tested for differential abundance and variability between cultivation conditions, while controlling for the effect of dilution and day on dispersion.

### Organic extractions and HPLC-ESI mass spectrometry

After overnight ethyl acetate extractions, the organic layer was transferred to round-bottom flasks and evaporated on a Heidolph Hei-Vap Value (Heidolph, Schwabach, Germany) rotovap. Samples were resuspended in 100% methanol and transferred to pre-weighed scintillation vials. Samples were dried under nitrogen gas at room temperature and weighed for final yield (mg). For this experiment, all beakers were brand-new from the same lot, rinsed with 100% methanol prior to use, and one beaker was incubated overnight with 103 mL of ethyl acetate to generate a glassware blank to attribute any metabolites in the metabolomics data to glassware alone. Once samples were dry, they were stored at −80 °C until experiment was complete. Each sample was resuspended to a final concentration of 11 mg/mL in 100% isopropanol containing 0.1 μM of sulfadimethoxine as an internal standard. A volume of 100 μL of each sample was transferred to a LCMS/MS 96-well plate and stored at −80 °C until it was run on the instrument. In addition to the internal standard, a pooled sample control containing 2 μL of every sample was also prepared. Lastly, 100% methanol blanks were run between each experimental sample.

HPLC separation of the samples was performed on an Agilent 1290 HPLC system (Agilent, Santa Clara, CA, USA) with a Waters ACQUITY UPLC BEH column (ODS-18; 2.1 × 100 mm; 1.7 μm particle size, Waters, Milford, MA, USA). The resulting eluent from the HPLC column was run on an Agilent 6545 accurate mass Q-TOF mass spectrometer with an electrospray ionization source operating in the positive mode (Agilent, Santa Clara, CA, USA) (see Supplementary Detailed Methods). Data was collected with Mass Hunter Acquisition software (B.08.00).

### Molecular networking and spectral library search in GNPS

Raw data obtained from the LCMS/MS instrument was converted to.mzXML files using msConvert within ProteoWizard v3 [59]. The mass spectrometry data were first processed with MZmine2 v2.51 [60]. Peaks were identified (MS1 noise cutoff: 1.0E2, MS2 noise cutoff: 1.0E1). Chromatograms were built using the ADAP chromatogram builder [61] and deconvolution was achieved via the local minimum algorithm. Isotopes were grouped and the feature table was aligned and filtered (see Supplementary Detailed Methods for parameters). Poor peaks were removed from the peak list (long tails or plateaus) and any feature found in a blank (methanol, glassware, or isopropanol) was removed from the feature table. Results were exported to GNPS [62], for feature-based molecular networking analysis [63] (see Supplementary Detailed Methods for parameters).

### Metabolomics data analysis and hypothesis testing

Principal coordinate analysis using Bray–Curtis distances and 95% confidence ellipses calculated from total ion current (TIC)-normalized feature (metabolite) table was used to visualize the composition of metabolites using the r package *phyloseq* v1.26.1 [51]. The molecular networks were visualized using Cytoscape software v3.8.1 [64]. To test for differences, a PERMANOVA and PERMDISP were used (as described above) to characterize differences between metabolite composition among samples. To test the hypothesis that structured environments yield a community that produces a richer pool of metabolites, we performed a Mann–Whitney *U* test (Wilcoxon rank sum test) with Benjamini-Hochberg correction using the r package *stats* [65]. We also used a Fisher’s exact test to confirm differences in unique/total metabolites for each condition. To characterize how metabolite diversity changes over time, a liner mixed effects model was fit using REML via the r package *lme4* lmer function [66]. The formula Metabolites ~ Condition * Day + *(1* | *Dilution)* was used to compare the change in metabolite diversity (richness) over time between shaking and static conditions. This formula treated inoculum size as a random variable to account for dispersion while Day and Condition were fixed effects. Lastly, well-annotated metabolites were grouped into molecular classes using the classification program ClassyFire [67] and TIC-normalized values were summed for each class of molecule to generate a heatmap in PRISM v9 (GraphPad Software, San Diego, CA, USA).

### Data deposition and job accessibility

Sequence data have been deposited at NCBI’s Sequence Read Archive database under accession number PRJNA732431.

Mass spectrometry data were deposited on MassIVE (MSV000086507). The molecular networking job can be publicly accessed at https://gnps.ucsd.edu/ProteoSAFe/status.jsp?task=e96bf8001f6d4518834ac6ba3742bd81. (Supplemental run containing samples from Day 0 can be accessed at https://gnps.ucsd.edu/ProteoSAFe/status.jsp?task=807cc96f3c5d4898aca64659484d86ea).

## Results

### Differences in microbial community structure were primarily driven by environmental structure

**Fig. 1:**
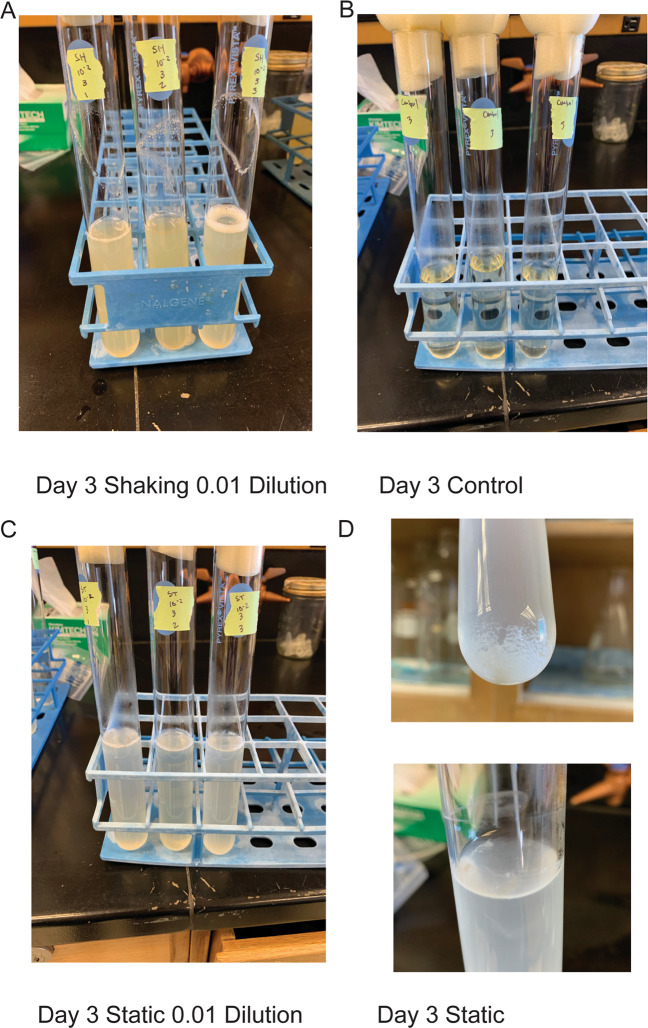
Images showing biofilm formation. Cultivation conditions included for **A** shaking, **B** controls, and **C** static cultures. Also shown are biofilms forming at the top or bottom of a static culture **D**.

**Fig. 2:**
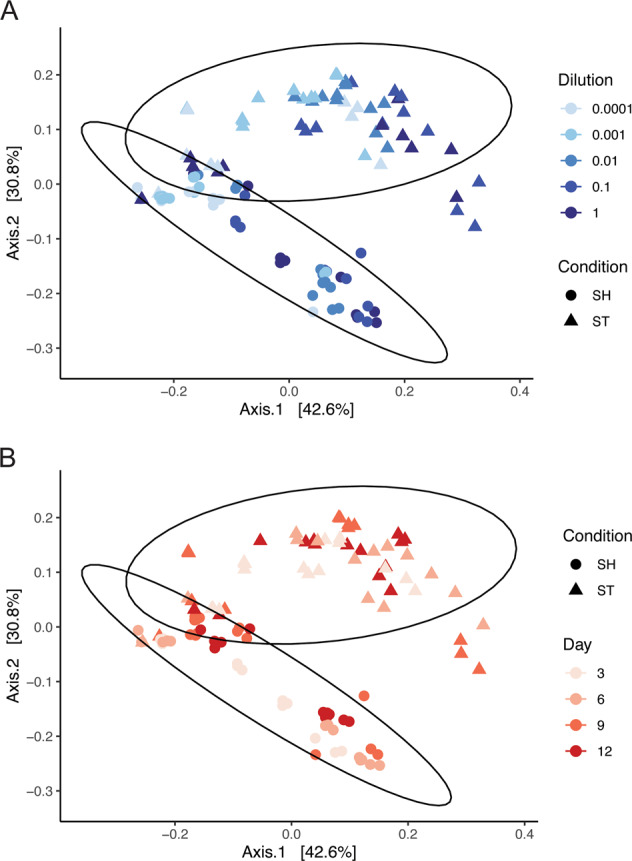
Principal coordinate analysis of microbial communities using weighted UniFrac distances. SH refers to shaking cultures, ST refers to static cultures. Shape and 95% confidence ellipses refer to cultivation condition, color refers to **A** inoculum size and **B** time. PERMANOVA cultivation condition: *F* = 75.121, *r*^2^ = 0.24, *p* value = 0.001. All PERMANOVA and PERMDISP results can be found in Supplementary Table 1A, B.

Serially diluted wastewater samples were incubated in shaking or static enrichments to represent unstructured or structured environments, respectively. The microbial diversity was characterized through 16S rRNA gene sequencing, while the chemical diversity was characterized through untargeted metabolomics. Despite the intention to remove structure through shaking and homogenization, many shaking cultures contained visible biofilm on the walls of the culture tube at the air-media interface (Fig. 1). However, there was nevertheless less visible structure in shaking cultures compared to static cultures. To focus on cultivated communities, Day 0 samples were removed from the analysis (See Supplementary Results and Supplementary Figs. 2–4). After removing Day 0, the most obvious pattern was the separation of static communities and shaking communities, where samples clustered according to cultivation condition (Fig. 2 and Supplementary Table 1A, B). Microbial communities also clustered according to inoculum size where samples partitioned from low to high dilutions for both static and shaking conditions (Fig. 2A). Lastly, microbial community structure changed little over time (Fig. 2B, Supplementary Table 1A, B). Interaction effects between variables suggest that main effects alone do not drive differences, but rather combinations of factors like time and condition jointly have an effect (Supplementary Table 1A, B). Interactions between treatments and the significant dispersion within treatments, like dilution or time, highlight important factors that will affect microbial community assembly. For most conditions, the beta diversity between replicate samples became more variable as inoculum size decreased (i.e., high variability for distance to centroid) (Supplementary Fig. 5A). Overall, beta diversity indicates that structure, afforded by static cultivation or removed by shaking cultivation, was the primary driver of diversity, though interactions among the factors show that the size of the starting community and time can influence community assembly in structured and non-structured environments as well.

### Structured environments selected for different community compositions

**Fig. 3:**
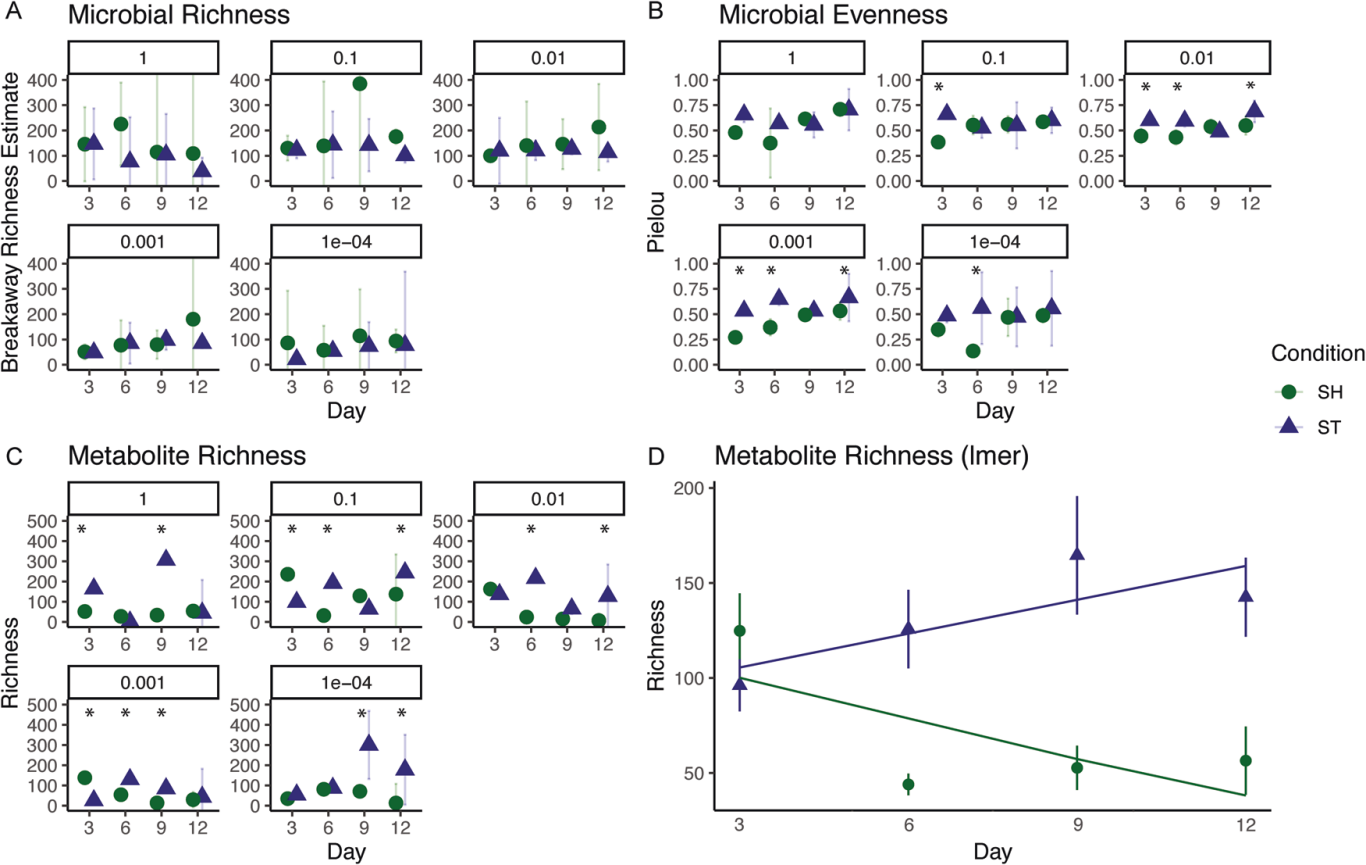
Microbial and metabolite alpha diversity metrics. Panels describe **A** microbial richness measured with breakaway estimates [52], **B** microbial evenness measured with Pielou’s Index [53], **C** metabolite richness, and **D** a mixed effects model of metabolite richness over time (Metabolites ~ Condition * Day + *(1* | *Dilution))*. Shape and color refer to cultivation condition. Data (**A**–**C**) were handled as independent samples based on inoculum size (*n* = 3), points are averages and bars are 95% confidence intervals. Significant differences from Tukey’s HSD test (*p* > 0.05) between shaking and static cultures designated with (*). All ANOVA tables and reporting can be found in the following Supplementary Tables: for microbial richness, Supplementary Tables 2–6; for microbial evenness, Supplementary Tables 7–11; and for metabolite richness, Supplementary Tables 13–17.

**Fig. 4:**
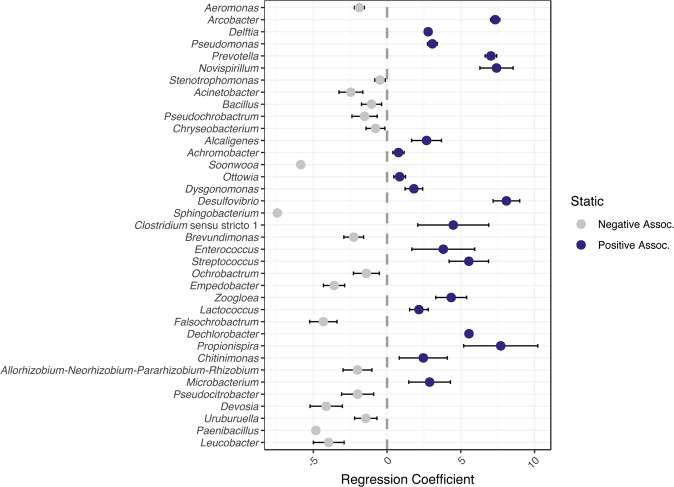
Differential abundance calculated using beta binomial regression at genus level between shaking and static conditions [58]. This model tested for differential abundance and variability between cultivation conditions, while controlling for the effect of dilution and day on dispersion. Blue refers to genera positively associated with static conditions; gray refers to genera negatively associated with static conditions. Order of genera based on relative abundance across experiment.

The number of different taxa (i.e., richness) was similar between shaking and static communities, and richness changed little over time (Fig. 3A, Supplementary Tables 2–6). In contrast, microbial evenness significantly differed between some shaking and static communities (Fig. 3B, Supplementary Tables 7–11). Microbial evenness also significantly changed over time (Fig. 3B, Supplementary Tables 7–11). Further, structured environments selected for higher abundances of certain taxa (Fig. 4), despite some taxa being shared between shaking and static conditions (Supplementary Fig. 6). Specifically, static cultures had significantly higher relative abundances of *Arcobacter* spp., *Prevotella* spp., *Pseudomonas* spp., and *Delftia sp*p. compared to shaking. We also observed lower abundant taxa, like *Desulfovibrio* spp., to have significantly higher relative abundances in static cultures compared to shaking cultures (Fig. 4). In contrast, shaking cultures were dominated by *Aeromonas* spp. with similar relative abundance across inoculum size (Supplementary Fig. 6). Overall, communities resulting from shaking or static samples were composed of similar populations but were structurally different.

### Cultivation conditions produced communities with different metabolic profiles

**Fig. 5:**
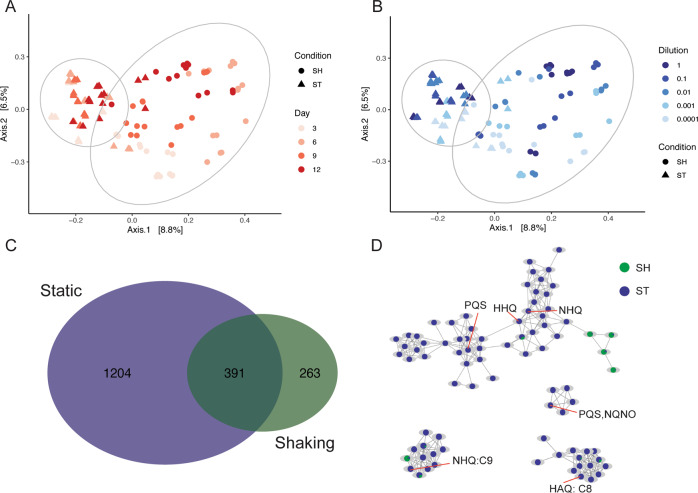
Principal Coordinate Analysis of metabolites using Bray–Curtis distances. Metabolite samples colored by **A** time and **B** inoculum size; shape and 95% CI refer to shaking (SH) or static (ST) PERMANOVA Cultivation Condition: *F* = 64.83, *r*^2^ = 0.07, *p* = 0.001. All PERMANOVA and PERMDISP results can be found in Supplementary Table 12A, B. **C** Venn diagram showing metabolites shared or unique between cultivation conditions. **D** Networks of Pseudomonad signaling metabolites. Nodes are pie charts showing the relative metabolite abundance between cultivation conditions. Orange call-out lines indicate an annotated node (see Supplementary Fig. 7 for mirror plots supporting annotations). HAQ refers to 4-hydroxy-2-octylquinolone 1-oxide. NHQ is 2-nonylquinolin-4(1H)-one. HHQ is 2-heptylquinolin-4(1H)-one. PQS is *Pseudomonas* quinolone signal. NQNO is 2-nonyl-4-hydroxyquinolone *N*-oxide [71].

Since this study focused on the effect of structured environments on microbial interactions, our untargeted metabolomics methods primarily characterized specialized and secondary metabolites, molecules typically driving these types of interaction [68]. We observed the structure of the metabolome to differ based on individual factors like cultivation condition, time, and inoculum size based on higher F-values, more so than interactions among factors. (Figs. 5A, B, Supplementary Table 12A, B). This indicates that these conditions (i.e., shaking or static) resulted in different metabolic profiles over time and based on the size of the starting inoculum. A total of 391 metabolites were shared between shaking and static cultures, while static cultures had 1204 unique metabolites and shaking cultures had 263 unique metabolites (Fisher’s exact test, *p* < 0.0001) (Fig. 5C). Additionally, static cultures had significantly higher richness than shaking cultures (Mann-Whitney/Wilcoxon, W = 952.5, *p* value = 3.657e−05), though differences in richness between shaking and static was dependent on starting inoculum sizes (Fig. 3C, Supplementary Tables 13–17). We also found an overall greater diversity of metabolites over time in static cultures (anova(lmer):Chi-sq = 22.4, *p* < 0.0001) (Fig. 3D), but not shaking cultures. Because we normalized by weight, the increase in metabolite counts is not simply due to increases in biomass as cultures grew.

**Fig. 6:**
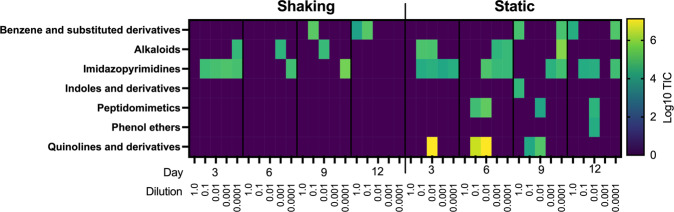
TIC-normalized quantifications of annotated metabolites were classified using ClassyFire [67]. Individual metabolite quantifications were summed based on class and log10-transformed to create a heatmap describing classes of annotated molecules.

Classes of molecules identified in the metabolome differed between shaking and static condition (Fig. 6). Of these metabolites that could be annotated, the majority belonged to families of compounds involved in quorum sensing and signaling, particularly observed in *Pseudomonas* spp. physiology (Figs. 5D, 6 and Supplementary Fig. 7). The production of these molecules and the ability to rapidly adapt make pseudomonads successful colonizers and aggressors [31, 69–71]. Lastly, compounds known to have antagonistic activity were more common in static cultures compared to shaking cultures, like the phenol ether, anisomycin [72, 73], or peptidomimetic orfamides [74–76] (Fig. 6, Supplementary Figs. 7 and 8). This observation suggests that structured environments afford more opportunity to interact and may select for organisms that use metabolite-mediated strategies to be successful during community assembly, compared to shaking cultures where the advantage was most probably due to increased growth rate (e.g. *Aeromonas* sp.).

## Discussion

By manipulating the physical environment through shaking or static cultivation, we characterized the microbial community response through changes in microbial diversity and chemodiversity. Our data show that (1) environmental structure drives differences in the metabolome and microbiome, (2) environmental structure selected for more evenness in microbial communities compared to unstructured environments, and (3) environmental structure selected for higher metabolite richness. We show that environmental structure plays a significant role in secondary metabolic processes that may drive community assembly. These findings add to efforts seeking to determine what drives microbial community assembly and how the metabolome can be linked to those drivers.

Community structure was primarily determined by cultivation condition, suggesting that selection was driven by the structure of the environment. Because there is no pattern of change in structure from day 3 through day 12 (Fig. 2), we suggest that cultivation condition has the greatest selective effect and benefited strains that could grow quickly. Constant mixing of the shaking cultures reduced any oxygen gradients and potentially selected for fast-growing taxa that would allocate resources to increase growth rate over costly secondary metabolite production, which would have a diminished local effect in well-mixed environments [12]. The suggestion is supported by the fact that *Aeromonas* sp. quickly establish dominance (Day 3) in shaking cultures compared to static cultures that had higher evenness. Static cultures could have provided different niches, particularly along an oxygen gradient, that may allow different taxa to grow equally well, resulting in more even communities [31]. This is contrasted with shaking cultures, that possibly selected for more oxygen-adapted taxa that were able to quickly outcompete other taxa, resulting in communities with a dominate taxon, like *Aeromonas* sp. However, this ubiquitous taxon, commonly found in wastewater [77, 78] and gastrointestinal tracts [79], is a known biofilm producer [39]. Despite the intention to remove structure, the shaking cultures yielded visible biofilms on the walls of the tubes where medium was washed through shaking (Fig. 1).

In contrast, static cultures exhibited significant biofilm formation but did not contain abundant *Aeromonas* spp. populations (Fig. 4). Of the taxa that were significantly associated with static cultures, *Arcobacter* spp., *Delftia* spp., and *Pseudomonas* spp. were the most abundant in those cultures. Each of these genera contain members involved in biofilm formation [80–82]. Further, *Pseudomonas* spp. are particularly associated with competitive interactions in structured environments, like biofilms [83, 84]. The act of a population outcompeting and establishing itself will create spatial structure [4], since a molecule acting locally will help generate structure in chemical space by establishing a gradient [4, 35]. In our experiment, we observed *Pseudomonas sp*. to be significantly associated with structured conditions and elicitation of multiple families of metabolites associated with pseudomonad quorum sensing and signaling (Figs. 5 and 6). Overall, cultivation conditions selected for differential abundances of shared taxa resulting in two biofilm-forming communities that either had a dominant taxon (shaking) or a more even community composition (static), highlighting the growth mechanism of microorganisms seeking to establish or thriving in structure [3, 29] through biomass and/or metabolite production [85–88]. Future efforts should seek to quantify the formation of a biofilm (e.g., stains of polymeric substances or fluorescence in situ hybridization (FISH) for specific taxa).

Differences in community structure were also affected by the size of the starting inoculum (i.e. dilution) (Fig. 2B). Diluting the inoculum reduced the number of cultivable organisms and introduced variability at high dilutions. The effect of decreased inoculum sizes has been demonstrated in dilution-to-extinction cultivation efforts [13]. We observed this in our data where, despite having differences in community structure based on dilution, we observed significant dispersion within each dilution (Supplementary Table 1B, Supplementary Fig. 5A). This would suggest that community assembly follows a predictable trend when the inoculum is larger, but as populations are diluted, the outcome of community assembly becomes more difficult to predict. Conversely, little dispersion between static and shaking samples suggests that the effect of environmental structure is acting in a predictable manner (Supplementary Fig. 5B). When samples vary differentially, this could indicate that the cultivation conditions may not be selecting for a single type of community or taxon through deterministic processes (e.g. interactions or environmental filtering) where they are acting equally on each dilution, but rather creating stochastic assembly opportunities, where the establishment of an organism is based on random, non-niche based chance [89] introduced through altering the inoculum size. In short, the success of an organism will be a function of its abundance in the inoculum where both stochastic and deterministic processes can affect that success.

Though environmental structure did not influence the richness of a microbial community, we did observe these cultivation conditions to select for a more even community. Evenness has been implicated in the resilience, stability, and productivity of microbial communities [90, 91]. We observed that more even communities had higher richness of metabolites, highlighting that microbiome-level metrics of richness failed to capture the metabolic capabilities of a community, particularly those related to interactions and the production of metabolites. One explanation for the differences in the metabolome between structured and non-structured environments could be that it was driven by the bacterial strains for which the conditions are selecting and the method through which the metabolites were extracted. For example, structured environments could be selecting for organisms that use structure and the production of small molecules as an advantage [15, 92], like *Pseudomonas* spp. during biofilm production [93, 94]. But it is important to acknowledge that non-polar extraction methods, like ethyl acetate used here, will favor the recovery of less polar molecules, like *Pseudomonas* spp. metabolites. In this case, both environmental structure and the type of molecules extracted would describe differences in the metabolome, rather than an effect driven by the microbial richness of that community.

Metabolome diversity (richness) increased over time and the structure was primarily driven by cultivation condition and time (Supplementary Table 12A). This increase in metabolite diversity could potentially highlight different metabolic mechanisms aiding populations during community assembly. For example, differences in the composition of the metabolome could be related to strategies for increased growth rates during community assembly where many of the detected metabolites are by-products of rapid resource consumption. Alternatively, in the case of our structured environment data, the composition of the metabolome had many molecules used during interactions, like quinolones and their derivatives [70, 71], (Figs. 5D, 6, Supplementary Figure 7) and/or had antimicrobial properties, like anisomycin and orfamide [72, 73, 75, 76] (Fig. 6, Supplementary Figs. 7 and 8). This suggests that biotic interaction played a role during community assembly in structured environments. The type of interaction can vary [95–97], but for those mediated by small diffusible molecules, the effects of those molecules will have greater effect in shorter ranges [12, 30, 98], something a structured environment would provide. Here, we showed that community assembly patterns are dependent on environmental structure, and that a structured environment selected for communities with higher chemical diversity. Not only do these findings seek to connect the ‘who is there’ and ‘what are they doing’ paradigm of ecology, but also highlights the role cultivation can play in targeting new metabolisms for natural product discovery.

## Supplementary information

Supplemental Material
