# Supplementary material for: Environmental structure impacts microbial composition and secondary metabolism
Source: ISME Commun. 2022 Feb 3;2:15. doi: 10.1038/s43705-022-00097-5 (PMC9723690; doi:10.1038/s43705-022-00097-5)
Supplement: Supplementary file 1 — Supplemental Material [file 43705_2022_97_MOESM1_ESM.pdf]

Supplemental Detailed Methods

Sampling and Experimental Set Up

Wastewater was collected from the aerator basin at the Norman Water Reclamation Facility, Norman, OK on 11 February 2020, stored on ice, and transported immediately back to the laboratory (< 30 minutes). The sample (50 mL volume) was homogenized vigorously on a vortex mixer for ~2 minutes to break up flocks and produce a slurry. The sample was serially diluted (1:10) in sterile R2B liquid medium four times to generate 5 different dilutions (dilution factors of 1 to 10 000) to be used for the starting inocula (Reasoner & Geldreich, 1985). Each dilution was inoculated into sterile R2B medium for a final volume of 35 mL. A total of 150 enrichments were made so that three replicates could be sampled destructively, representing each dilution (1E-00, 1E-01, 1E-02, 1E-03, 1E-04) for each sampling day (Day 0, 3, 6, 9, 12) and for each condition (shaking or static). A total of 15 uninoculated medium controls were made to be used at each time point (n=3); these were incubated with the static samples. All cultures were incubated at 30 °C, either shaking at 250 rpm or static (Supplemental Figure 1).

On each sampling day (0, 3, 6, 9, 12) prior to DNA extraction, triplicate cultures from each inoculum size and condition were vortexed and pooled into a sterile glass beaker. Any remaining biofilm was scraped from the glass walls of each test tube using a sterile, silicon cell scraper (Sarstedt, Nümbrecht, Germany), transferred to the sterile beaker, and mixed. From this pool, three 1.0 mL aliquots were transferred to sterile micro centrifuge tubes and spun at 10,000 x g for 1 min. The supernatant was removed, cell pellets were resuspended in 1 mL of sterile 1x PBS and spun again at 10 000 x g for 1 min to generate a cell pellet. After the 1x PBS supernatant was removed, cells were resuspended in 750 uL Zymo bashing bead buffer (per

Zymo Quick DNA manufacturer protocol), transferred to Zymo bashing bead tubes (Zymo Research Corp., Irvine, CA, USA), homogenized for 45 seconds using a hand-held reciprocating saw with custom attachment (RYOBI, Anderson, SC, USA), and stored at -20 °C until DNA extraction. Lastly, 103 mL of ethyl acetate was added to the remaining volume of the pooled samples (~103 mL) and mixed to generate a 1:1 ratio of culture and solvent for an overnight, organic extraction at room temperature. Medium controls for each time point, described above, were pooled, sampled for DNA extraction, and prepared for ethyl acetate organic extraction as described above for the cultures to be used as blanks for both sequencing and metabolomic data.

HPLC-ESI Mass Spectrometry

A 5 µL volume at 11 mg/mL was used for injection on an Agilent 1290 HPLC system (Agilent, Santa Clara, CA, USA) with a Waters ACQUITY UPLC BEH column (ODS-18; 2.1 x 100 mm; 1.7 µm particle size, Waters, Milford, MA, USA). Mobile phase A was comprised of 0.1% formic acid in Optima LC/MS water (FisherScientific, Waltham, MA, USA), while mobile phase B was comprised of 0.1% formic acid in 100% HPLC-grade acetonitrile. A binary gradient at 0.4 mL/min flow rate was used under the following steps: 90% solvent A and 10% solvent B from 0 to 1 min, linear gradient to 20% solvent B from 1-2 min, linear gradient to 80% solvent B from 2-16 min, linear gradient to 100% solvent B from 16-18 min, and 100% solvent B from 20 – 21 min, which was maintained for an additional 2 min before the next sample injection. Eluent from the column was run on an Agilent 6545 accurate mass Q-TOF mass spectrometer with an electrospray ionization source operating in the positive mode (Agilent, Santa Clara, CA, USA). Nitrogen was used as a nebulizing gas (40 lbs/in^2) and as a drying gas (325 °C; 10 L/min flow rate) (Bartley et al., 2013). Fragmentor voltage was 180 V, skimmer voltage was 45 V, and

capillary voltage was 4,000 V. Untargeted MS/MS was used with mass-dependent collision energy ramp from 20-35 V using ultra high purity nitrogen. Data was collected with Mass Hunter Acquisition software (B.08.00).

Molecular Networking and Spectral Library Search in GNPS

Raw data obtained from the LCMS/MS instrument was converted to .mzXML files using msConvert within ProteoWizard v3 (Chambers et al., 2012). The mass spectrometry data were first processed with MZmine2 v2.51 (Pluskal et al., 2010). Peaks were identified (MS1 noise cutoff: 1.0E2, MS2 noise cutoff: 1.0E1). Chromatograms were built under the following criteria using the ADAP chromatogram builder (Myers et al., 2017): MS1 scans; Minimum Group Number: 5; Group Intensity: 2.0E2; Minimum High Intensity: 1.0E3; *m/z* tolerance: 0.02 *m/z* or 0.0 ppm. Deconvolution was achieved through the following parameters via the local minimum algorithm: Chromatographic Threshold: 5.0%; search minimum in RT range: 0.4 min; Minimum Relative Height: 5.0%; Minimum Absolute Height: 2.0E3; Minimum Ratio Peak Top/Edge: 1; Duration: 0.01-3.50 min; Algorithm: Median; *m/z* MS2: 0.01Da; RT Range: 0.2 min. Isotopes were grouped under the following criteria: *m/z* total: 0.02; RT total: 0.10 min; Maximum charge: 3; Representative Isotope: Max Intensity. The feature table was constructed with using the following alignment parameters: *m/z* tolerance: 0.02 *m/z* or 200 ppm; Weight for *m/z*: 75; RT tolerance: 0.1 min; Weight for RT: 25 and filtered using the following criteria: Min peaks in per row : 3; Keep only peaks with MS2: yes. Poor peaks were removed from the peak list (long tails or plateaus) and any feature found in a blank (methanol, glassware, or isopropanol) was removed from the feature table. Results were exported to GNPS (M. Wang et al., 2017), for feature based molecular networking (FBMN) analysis (Nothias et al., 2020).

In GNPS the data were analyzed using FBMN workflow (Nothias et al., 2020). The data were filtered by removing all MS/MS fragment ions within +/- 17 Da of the precursor *m/z*. MS/MS spectra were filtered by choosing the top 6 fragment ions in the +/- 50 Da window. The precursor ion mass tolerance was set to 0.02 Da and the MS/MS fragment ion tolerance to 0.02 Da. A molecular network was created with edges (filtered via cosine score > 0.7 and > 4 matched peaks). Also, edges between nodes were kept only if each of the nodes appeared in each other's respective top 10 most similar nodes. Finally, the maximum size of a molecular family was set to 100. The analogue search mode searched against MS/MS spectra with a maximum difference of 100 Da in the precursor ion value. The library spectra were filtered as the input data was filtered, as discussed above, cosine score above 0.7 and at least 4 matched peaks.

Microbial and Metabolite Richness Correlations

To investigate the relationship between microbial and metabolite alpha diversity measures, we performed a Spearman's rank-based correlation comparing the metabolite richness to each alpha diversity measure using the r packages *Hmisc* and *corrplot* (Frank E Harrell Jr, 2020; Wei & Simko, 2017)

Supplemental Results

Day 0 beta diversity

Differences in community structure were primarily driven by inoculum size (PERMANOVA F= 18.97, r^2= 0.18, p=0.001; PERMDISP F=0.71, p=0.599). Both cultivation condition and day significantly contributed to community structure. However, within-group variance was high and could affect the significance contribution to community structure. Day 0 samples were tightly clustered, indicating high similarity between Day 0 samples, which was expected (Supplemental Figure 2). Because Day 0 samples represented the starting community that had not undergone any selection for which we were testing and would thus skew the distance matrix, they were removed for subsequent analyses. Like the sequence data, Day 0 metabolites clustered tightly and away from the rest of the data set. Because these metabolites represent what was in the inoculum before any selection would take place based on our hypothesis, these samples were removed from subsequent analysis (Supplemental Figure 3).

Microbial and Metabolite Richness Correlations

We initially hypothesized that structured environments would have higher microbial richness as a function of environmental gradients creating diversified niche-availability and would also have higher richness of metabolites as a function of higher richness of community members. To investigate this relationship, we performed a Spearman's correlation on the richness and evenness of the microbial community and the metabolomes. We observed that metabolite richness had no significant correlations with microbial alpha diversity measures (breakaway/microbial richness: r^2= -0.02, p= 0.88; microbial evenness: r^2= 0.23, p= 0.16, metabolite evenness : r^2= -0.04, p = 0.83) (Supplemental Figure 4). This suggests that it may not

be the number of organisms in a community that matter for metabolite diversity, but rather who they are and the strategies they employ during community assembly, like pseudomonads and the fleet of metabolites they produce, compared to *Aeromonas* sp., that quickly outgrew other populations.

Supplemental Tables

Supplemental Table 1 contains the PERMANOVA and PERMDISP results for microbial beta diversity comparisons.

Supplemental Table 1a: PERMANOVA of weighted UniFrac microbial beta diversity (n=114). (~Dilution*Condition*Day, permutations = 999).						
	DF	Sum of Sqs.	Mean Sqs	F	r^2	p value
Dilution	4	1.2330	0.30824	13.888	0.17459	0.001
Condition	1	1.6673	1.66734	75.121	0.23610	0.001
Day	3	0.4275	0.14251	6.421	0.06054	0.001
Dilution:Condition	4	0.3766	0.09415	4.242	0.05333	0.001
Dilution:Day	12	0.7099	0.05916	2.665	0.10052	0.001
Condition:Day	3	0.2485	0.08282	3.732	0.03518	0.001
Dil:Con:Day	12	0.7567	0.06306	2.841	0.10716	0.001
Residuals	74	1.6425	0.02220		0.23258	
Total	113	7.0620			1.00000	

Supplemental Table 1b: PERMDISP of weighted UniFrac microbial beta diversity (n=114).						
Permutation test for homogeneity of multivariate dispersions for Condition						
	DF	Sum of Sqs.	Mean Sqs	F	N perm	p value
Groups	1	0.00749	0.0074883	1.4608	999	p=0.218
Residuals	112	0.57412	0.0051261			
Permutation test for homogeneity of multivariate dispersions for Dilution						
	DF	Sum of Sqs.	Mean Sqs	F	N perm	p value
Groups	4	0.0492	0.0123003	2.7113	999	p=0.038
Residuals	109	0.4945	0.0045367			
Permutation test for homogeneity of multivariate dispersions for Day						
	DF	Sum of Sqs.	Mean Sqs	F	N perm	p value
Groups	3	0.07773	0.0259087	4.8044	999	p=0.007
Residuals	110	0.59320	0.0053927			

Supplemental tables 2-6 contain the two-way ANOVA results and Tukey's HSD multiple comparisons for microbial richness estimates.

Supplemental Table 2a: Dilution Factor 1 (10^0) ANOVA tables for microbial richness. Tests run in PRISM v 9.1.0 (GraphPad).					
ANOVA	SS	DF	MS	F (DFn, DFd)	P value
Interaction	20545	3	6848	F (3, 14) = 1.5	P=0.2655
Row Factor (Condition)	17427	1	17427	F (1, 14) = 3.7	P=0.0736
Column Factor (Time)	20534	3	6845	F (3, 14) = 1.5	P=0.2657
Residual	65240	14	4660		

Supplemental Table 2b: Dilution Factor 1 (10^0) multiple comparisons tests (Tukey's HSD) for microbial richness between shaking and static conditions. Tests run in PRISM v 9.1.0 (GraphPad). Significance reported in Figure 3A (*).		
Tukey's multiple comparisons test	q	P Value
SH:3 0 vs. ST:3 0	0.027	>0.9999
SH:6 0 vs. ST:6 0	3.8	0.2095
SH:9 0 vs. ST:9 0	0.23	>0.9999
SH:12 0 vs. ST:12 0	1.6	0.9380

Supplemental Table 3a: Dilution Factor 0.1 (10^{-1}) ANOVA tables for microbial richness. Tests run in PRISM v 9.1.0 (GraphPad).					
ANOVA	SS	DF	MS	F (DFn, DFd)	P value
Interaction	56134	3	18711	F (3, 15) = 1.3	P=0.2972
Row Factor (Condition)	36038	1	36038	F (1, 15) = 2.6	P=0.1283
Column Factor (Time)	73714	3	24571	F (3, 15) = 1.8	P=0.1966
Residual	208616	15	13908		

Supplemental Table 3b: Dilution Factor 0.1 (10^{-1}) multiple comparisons tests (Tukey's HSD) for microbial richness between shaking and static conditions. Tests run in PRISM v 9.1.0 (GraphPad). Significance reported in Figure 3A (*).		
Tukey's multiple comparisons test	q	P Value
SH:3 -1 vs. ST:3 -1	0.11	>0.9999
SH:6 -1 vs. ST:6 -1	0.063	>0.9999
SH:9 -1 vs. ST:9 -1	3.6	0.2585
SH:12 -1 vs. ST:12 -1	1.1	0.9922

Supplemental Table 4a: Dilution Factor 0.01 (10^{-2}) ANOVA tables for microbial richness. Tests run in PRISM v 9.1.0 (GraphPad).					
ANOVA	SS	DF	MS	F (DFn, DFd)	P value
Interaction	11263	3	3754	F (3, 15) = 1.9	P=0.1662
Row Factor (Condition)	5020	1	5020	F (1, 15) = 2.6	P=0.1279
Column Factor (Time)	8846	3	2949	F (3, 15) = 1.5	P=0.2488
Residual	28996	15	1933		

Supplemental Table 4b: Dilution Factor 0.01 (10^{-2}) multiple comparisons tests (Tukey's HSD) for microbial richness between shaking and static conditions. Tests run in PRISM v 9.1.0 (GraphPad). Significance reported in Figure 3A (*).		
Tukey's multiple comparisons test	q	P Value
SH:3 -2 vs. ST:3 -2	0.77	0.9991
SH:6 -2 vs. ST:6 -2	0.78	0.9990
SH:9 -2 vs. ST:9 -2	0.68	0.9996
SH:12 -2 vs. ST:12 -2	3.9	0.1730

Supplemental Table 5a: Dilution Factor 0.001 (10^{-3}) ANOVA tables for microbial richness. Tests run in PRISM v 9.1.0 (GraphPad).					
ANOVA	SS	DF	MS	F (DFn, DFd)	P value
Interaction	9743	3	3248	F (3, 16) = 1.2	P=0.3583
Row Factor (Condition)	4417	1	4417	F (1, 16) = 1.6	P=0.2285
Column Factor (Time)	21059	3	7020	F (3, 16) = 2.5	P=0.0973
Residual	45074	16	2817		

Supplemental Table 5b: Dilution Factor 0.001 (10^{-3}) multiple comparisons tests (Tukey's HSD) for microbial richness between shaking and static conditions. Tests run in PRISM v 9.1.0 (GraphPad). Significance reported in Figure 3A (*).		
Tukey's multiple comparisons test	q	P Value
SH:3 -3 vs. ST:3 -3	0.10	>0.9999
SH:6 -3 vs. ST:6 -3	0.26	>0.9999
SH:9 -3 vs. ST:9 -3	0.60	0.9998
SH:12 -3 vs. ST:12 -3	3.1	0.4042

Supplemental Table 6a: Dilution Factor 0.0001 (10^{-4}) ANOVA tables for microbial richness. Tests run in PRISM v 9.1.0 (GraphPad).					
ANOVA	SS	DF	MS	F (DFn, DFd)	P value
Interaction	8555	3	2852	F (3, 14) = 1.2	P=0.3364
Row Factor (Condition)	653	1	653	F (1, 14) = 0.28	P=0.6043
Column Factor (Time)	6988	3	2329	F (3, 14) = 1.0	P=0.4204
Residual	32511	14	2322		

Supplemental Table 6b: Dilution Factor 0.0001 (10^{-4}) multiple comparisons tests (Tukey's HSD) for microbial richness between shaking and static conditions. Tests run in PRISM v 9.1.0 (GraphPad). Significance reported in Figure 3A (*).		
Tukey's multiple comparisons test	q	P Value
SH:3 -4 vs. ST:3 -4	2.3	0.7125
SH:6 -4 vs. ST:6 -4	0.10	>0.9999
SH:9 -4 vs. ST:9 -4	1.5	0.9596
SH:9 -4 vs. ST:12 -4	0.12	>0.9999
SH:12 -4 vs. ST:12 -4	0.54	>0.9999

Supplemental tables 7-11 contain the two-way ANOVA results and Tukey's HSD multiple comparisons for microbial evenness measured in Pielou's Index.

Supplemental Table 7a: Dilution Factor 1 (10^{0}) ANOVA tables for microbial evenness. Tests run in PRISM v 9.1.0 (GraphPad).					
ANOVA	SS	DF	MS	F (DFn, DFd)	P value
Interaction	0.11	3	0.035	F (3, 14) = 5.1	P=0.0137
Row Factor (Condition)	0.0022	1	0.0022	F (1, 14) = 0.32	P=0.5829
Column Factor (Time)	0.15	3	0.049	F (3, 14) = 7.1	P=0.0040
Residual	0.097	14	0.0070		

Supplemental Table 7b: Dilution Factor 1 (10^0) multiple comparisons tests (Tukey's HSD) for microbial evenness between shaking and static conditions. Tests run in PRISM v 9.1.0 (GraphPad). Significance reported in Figure 3B (*).		
Tukey's multiple comparisons test	q	P Value
SH:3 0 vs. ST:3 0	3.7	0.2344
SH:6 0 vs. ST:6 0	4.0	0.1548
SH:9 0 vs. ST:9 0	1.1	0.9919
SH:12 0 vs. ST:12 0	0.076	>0.9999

Supplemental Table 8a: Dilution Factor 0.1 (10^{-1}) ANOVA tables for microbial evenness. Tests run in PRISM v 9.1.0 (GraphPad).					
ANOVA	SS	DF	MS	F (DFn, DFd)	P value
Interaction	0.079	3	0.026	F (3, 15) = 14	P=0.0001
Row Factor (Condition)	0.033	1	0.033	F (1, 15) = 18	P=0.0008
Column Factor (Time)	0.016	3	0.0053	F (3, 15) = 2.8	P=0.0749
Residual	0.028	15	0.0019		

Supplemental Table 8b: Dilution Factor 0.1 (10^{-1}) multiple comparisons tests (Tukey's HSD) for microbial evenness between shaking and static conditions. Tests run in PRISM v 9.1.0 (GraphPad). Significance reported in Figure 3B (*).		
Tukey's multiple comparisons test	q	P Value
SH:3 -1 vs. ST:3 -1	11	<0.0001
SH:6 -1 vs. ST:6 -1	0.96	0.9963
SH:9 -1 vs. ST:9 -1	0.41	>0.9999
SH:12 -1 vs. ST:12 -1	0.59	0.9998

Supplemental Table 9a: Dilution Factor 0.01 (10^{-2}) ANOVA tables for microbial evenness. Tests run in PRISM v 9.1.0 (GraphPad).					
ANOVA	SS	DF	MS	F (DFn, DFd)	P value
Interaction	0.039	3	0.013	F (3, 15) = 13	P=0.0002
Row Factor (Condition)	0.058	1	0.058	F (1, 15) = 56	P<0.0001
Column Factor (Time)	0.046	3	0.015	F (3, 15) = 15	P<0.0001
Residual	0.016	15	0.0010		

Supplemental Table 9b: Dilution Factor 0.01 (10^{-2}) multiple comparisons tests (Tukey's HSD) for microbial evenness between shaking and static conditions. Tests run in PRISM v 9.1.0 (GraphPad). Significance reported in Figure 3B (*).		
Tukey's multiple comparisons test	q	P Value
SH:3 -2 vs. ST:3 -2	8.3	0.0006
SH:6 -2 vs. ST:6 -2	8.7	0.0004
SH:9 -2 vs. ST:9 -2	2.4	0.6841
SH:12 -2 vs. ST:12 -2	7.5	0.0018

Supplemental Table 10a: Dilution Factor 0.001 (10^{-3}) ANOVA tables for microbial evenness. Tests run in PRISM v 9.1.0 (GraphPad).					
ANOVA	SS	DF	MS	F (DFn, DFd)	P value
Interaction	0.058	3	0.019	F (3, 16) = 12	P=0.0002
Row Factor (Condition)	0.19	1	0.19	F (1, 16) = 123	P<0.0001
Column Factor (Time)	0.12	3	0.039	F (3, 16) = 25	P<0.0001
Residual	0.025	16	0.0016		

Supplemental Table 10b: Dilution Factor 0.001 (10^{-3}) multiple comparisons tests (Tukey's HSD) for microbial evenness between shaking and static conditions. Tests run in PRISM v 9.1.0 (GraphPad). Significance reported in Figure 3B (*).		
Tukey's multiple comparisons test	q	P Value
SH:3 -3 vs. ST:3 -3	11	<0.0001
SH:6 -3 vs. ST:6 -3	12	<0.0001
SH:9 -3 vs. ST:9 -3	1.8	0.9019
SH:12 -3 vs. ST:12 -3	5.8	0.0143

Supplemental Table 11a: Dilution Factor 0.0001 (10^{-4}) ANOVA tables for microbial evenness. Tests run in PRISM v 9.1.0 (GraphPad).					
ANOVA	SS	DF	MS	F (DFn, DFd)	P value
Interaction	0.13	3	0.043	F (3, 14) = 5.3	P=0.0118
Row Factor (Condition)	0.13	1	0.13	F (1, 14) = 17	P=0.0011
Column Factor (Time)	0.082	3	0.027	F (3, 14) = 3.4	P=0.0491
Residual	0.11	14	0.0081		

Supplemental Table 11b: Dilution Factor 0.0001 (10^{-4}) multiple comparisons tests (Tukey's HSD) for microbial evenness between shaking and static conditions. Tests run in PRISM v 9.1.0 (GraphPad). Significance reported in Figure 3B (*).		
Tukey's multiple comparisons test	q	P Value
SH:3 -4 vs. ST:3 -4	2.7	0.5833
SH:6 -4 vs. ST:6 -4	7.3	0.0028
SH:9 -4 vs. ST:9 -4	0.075	>0.9999
SH:12 -4 vs. ST:12 -4	1.2	0.9861

Supplemental Table 12 contains the PERMANOVA and PERMDISP results for metabolite beta diversity comparisons.

Supplemental Table 12a: PERMANOVA of Bray-Curtis metabolite beta diversity (n=114). (~Dilution*Condition*Day, permutations = 999).						
	DF	Sum of Sqs.	Mean Sqs	F	r^2	p value
Dilution	4	6.172	1.5431	26.737	0.11737	0.001
Condition	1	3.741	3.7414	64.829	0.07115	0.001
Day	3	5.342	1.7806	30.852	0.10158	0.001
Dilution:Condition	4	5.600	1.4000	24.258	0.10649	0.001
Dilution:Day	12	12.164	1.0136	17.563	0.23130	0.001
Condition:Day	3	4.042	1.3474	23.347	0.07687	0.001
Dil:Con:Day	12	11.082	0.9235	16.002	0.21074	0.001
Residuals	77	4.444	0.0577		0.08450	
Total	116	52.588			1.00000	

Supplemental Table 12b: PERMDISP of Bray-Curtis metabolite beta diversity (n=117).						
Permutation test for homogeneity of multivariate dispersions for Condition						
	DF	Sum of Sqs.	Mean Sqs	F	N perm	p value
Groups	1	0.033131	0.033131	18.444	999	p=0.001
Residuals	115	0.206581	0.001796			
Permutation test for homogeneity of multivariate dispersions for Dilution						
	DF	Sum of Sqs.	Mean Sqs	F	N perm	p value
Groups	4	0.007396	0.0018490	1.0408	999	p=0.395
Residuals	112	0.198960	0.0017764			
Permutation test for homogeneity of multivariate dispersions for Day						
	DF	Sum of Sqs.	Mean Sqs	F	N perm	p value
Groups	3	0.02133	0.0071102	2.847	999	p=0.041
Residuals	113	0.28221	0.0024974			

Supplemental tables 13-17 contain the two-way ANOVA results and Tukey's HSD multiple comparisons for metabolite richness.

Supplemental Table 13a: Dilution Factor 1 (10^0) ANOVA tables for metabolite richness (TIC normalized). Tests run in PRISM v 9.1.0 (GraphPad).					
ANOVA	SS	DF	MS	F (DFn, DFd)	P value
Interaction	85766	3	28589	F (3, 16) = 52	P<0.0001
Row Factor (Condition)	45938	1	45938	F (1, 16) = 84	P<0.0001
Column Factor (Time)	83965	3	27988	F (3, 16) = 51	P<0.0001
Residual	8797	16	550		

Supplemental Table 13b: Dilution Factor 1 (10^0) multiple comparisons tests (Tukey's HSD) for metabolite richness (TIC normalized) between shaking and static conditions. Tests run in PRISM v 9.1.0 (GraphPad). Significance reported in Figure 3C (*).		
Tukey's multiple comparisons test	q	P Value
SH:3 0 vs. ST:3 0	8.4	0.0004
SH:6 0 vs. ST:6 0	2.0	0.8479
SH:9 0 vs. ST:9 0	20	<0.0001
SH:12 0 vs. ST:12 0	0.66	0.9997

Supplemental Table 14a: Dilution Factor 0.1 (10^{-1}) ANOVA tables for metabolite richness (TIC normalized). Tests run in PRISM v 9.1.0 (GraphPad).					
ANOVA	SS	DF	MS	F (DFn, DFd)	P value
Interaction	88823	3	29608	F (3, 16) = 37	P<0.0001
Row Factor (Condition)	1601	1	1601	F (1, 16) = 2.0	P=0.1775
Column Factor (Time)	35547	3	11849	F (3, 16) = 15	P<0.0001
Residual	12871	16	804		

Supplemental Table 14b: Dilution Factor 0.1 (10^{-1}) multiple comparisons tests (Tukey's HSD) for metabolite richness (TIC normalized) between shaking and static conditions. Tests run in PRISM v 9.1.0 (GraphPad). Significance reported in Figure 3C (*).		
Tukey's multiple comparisons test	q	P Value
SH:3 -1 vs. ST:3 -1	8.4	0.0004
SH:6 -1 vs. ST:6 -1	9.8	<0.0001
SH:9 -1 vs. ST:9 -1	3.9	0.1689
SH:12 -1 vs. ST:12 -1	6.5	0.0055

Supplemental Table 15a: Dilution Factor 0.01 (10^{-2}) ANOVA tables for metabolite richness (TIC normalized). Tests run in PRISM v 9.1.0 (GraphPad).					
ANOVA	SS	DF	MS	F (DFn, DFd)	P value
Interaction	39116	3	13039	F (3, 15) = 23	P<0.0001
Row Factor (Condition)	40282	1	40282	F (1, 15) = 72	P<0.0001
Column Factor (Time)	43132	3	14377	F (3, 15) = 26	P<0.0001
Residual	8335	15	556		

Supplemental Table 15b: Dilution Factor 0.01 (10^{-2}) multiple comparisons tests (Tukey's HSD) for metabolite richness (TIC normalized) between shaking and static conditions. Tests run in PRISM v 9.1.0 (GraphPad). Significance reported in Figure 3C (*).		
Tukey's multiple comparisons test	q	P Value
SH:3 -2 vs. ST:3 -2	1.9	0.8652
SH:6 -2 vs. ST:6 -2	14	<0.0001
SH:9 -2 vs. ST:9 -2	3.7	0.2177
SH:12 -2 vs. ST:12 -2	7.9	0.0010

Supplemental Table 16a: Dilution Factor 0.001 (10^{-3}) ANOVA tables for metabolite richness (TIC normalized). Tests run in PRISM v 9.1.0 (GraphPad).					
ANOVA	SS	DF	MS	F (DFn, DFd)	P value
Interaction	34556	3	11519	F (3, 15) = 27	P<0.0001
Row Factor (Condition)	813	1	813	F (1, 15) = 1.9	P=0.1862
Column Factor (Time)	11482	3	3827	F (3, 15) = 9.0	P=0.0012
Residual	6355	15	424		

Supplemental Table 16b: Dilution Factor 0.001 (10^{-3}) multiple comparisons tests (Tukey's HSD) for metabolite richness (TIC normalized) between shaking and static conditions. Tests run in PRISM v 9.1.0 (GraphPad). Significance reported in Figure 3C (*).		
Tukey's multiple comparisons test	q	P Value
SH:3 -3 vs. ST:3 -3	9.5	0.0002
SH:6 -3 vs. ST:6 -3	6.4	0.0073
SH:9 -3 vs. ST:9 -3	6.0	0.0128
SH:12 -3 vs. ST:12 -3	1.0	0.9953

Supplemental Table 17a: Dilution Factor 0.0001 (10^{-4}) ANOVA tables for metabolite richness (TIC normalized). Tests run in PRISM v 9.1.0 (GraphPad).					
ANOVA	SS	DF	MS	F (DFn, DFd)	P value
Interaction	53082	3	17694	F (3, 15) = 14	P=0.0001
Row Factor (Condition)	62012	1	62012	F (1, 15) = 48	P<0.0001
Column Factor (Time)	63762	3	21254	F (3, 15) = 17	P<0.0001
Residual	19229	15	1282		

Supplemental Table 17b: Dilution Factor 0.0001 (10^{-4}) multiple comparisons tests (Tukey's HSD) for metabolite richness (TIC normalized) between shaking and static conditions. Tests run in PRISM v 9.1.0 (GraphPad). Significance reported in Figure 3C (*).		
Tukey's multiple comparisons test	q	P Value
SH:3 -4 vs. ST:3 -4	0.94	0.9969
SH:6 -4 vs. ST:6 -4	0.29	>0.9999
SH:9 -4 vs. ST:9 -4	11	<0.0001
SH:12 -4 vs. ST:12 -4	7.1	0.0028

Supplemental Figures

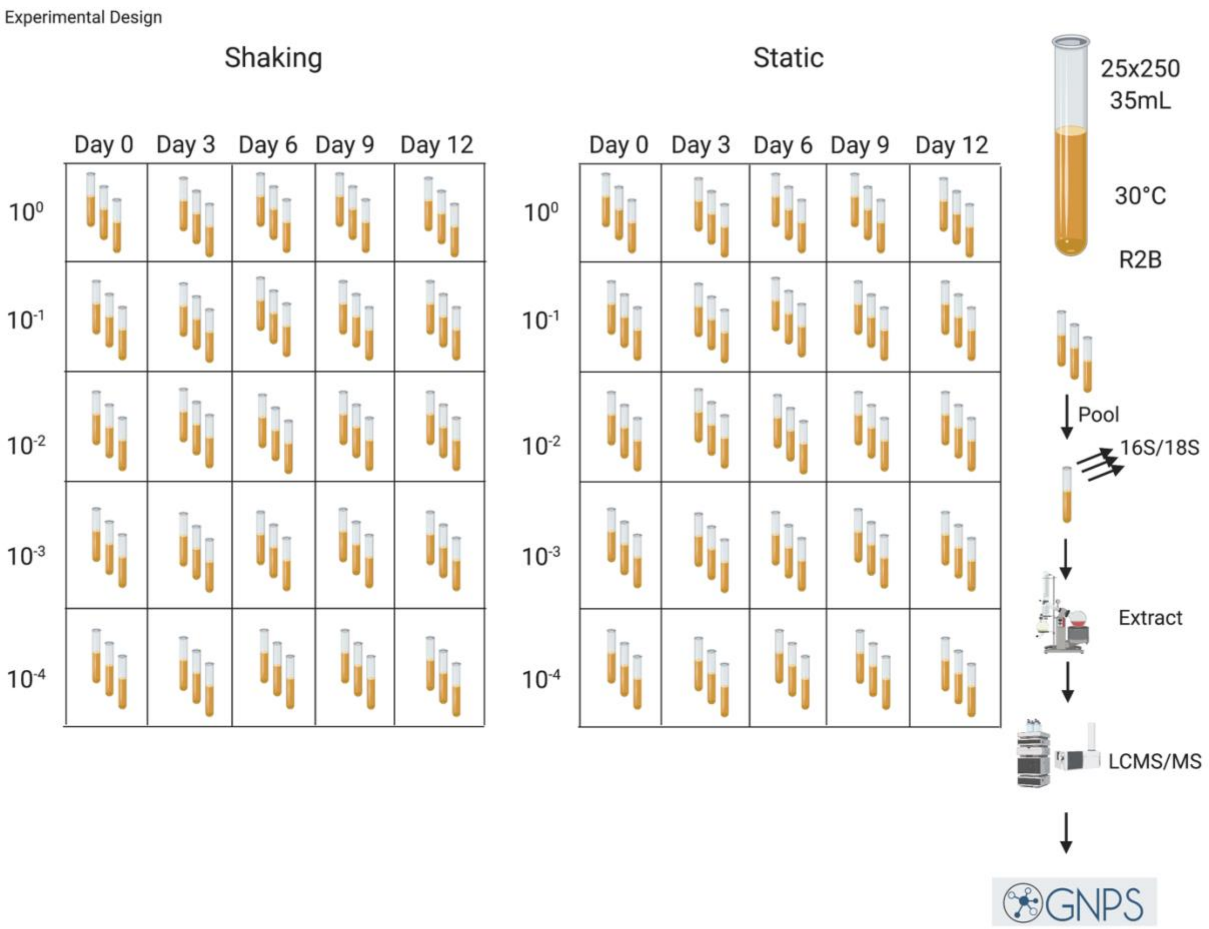

Supplemental Figure 1: Schematic of experimental setup. Created with BioRender.com.

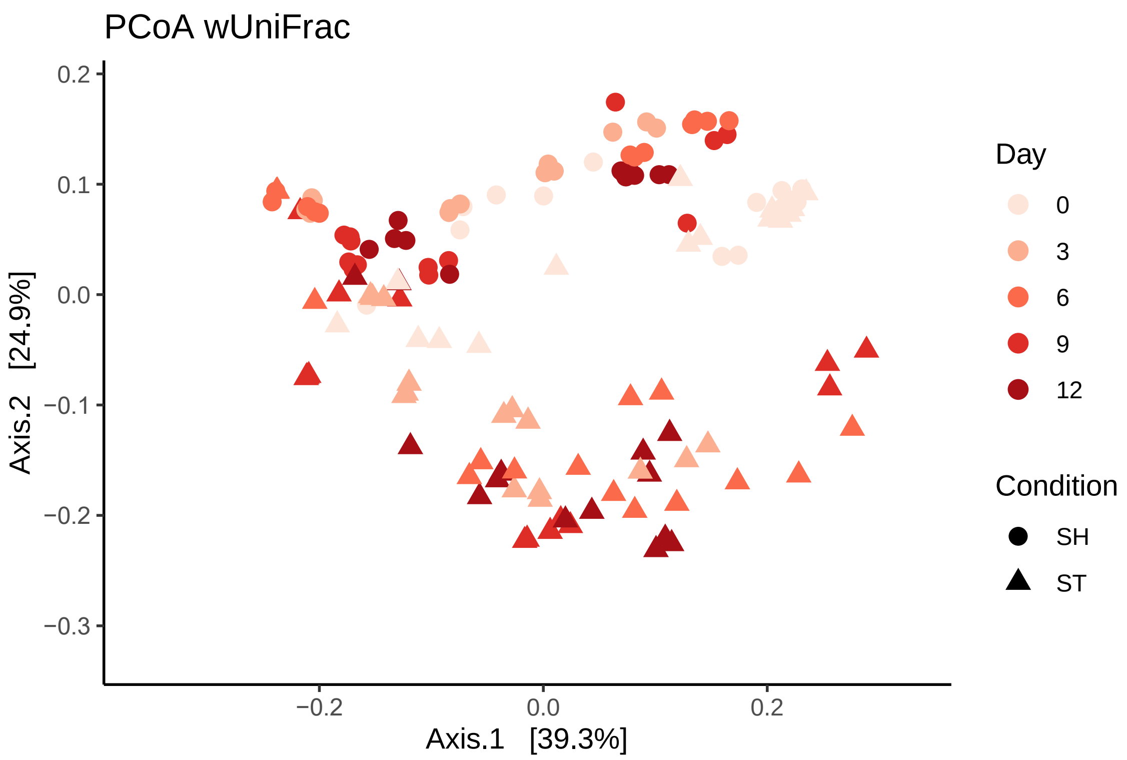

Supplemental Figure 2: Principal Coordinate Analysis of microbial communities including Day 0 samples using weighted UniFrac distances. Shape and 95% confidence interval ellipses refer to cultivation condition, color refers to time. SH refers to shaking, ST refers to static.

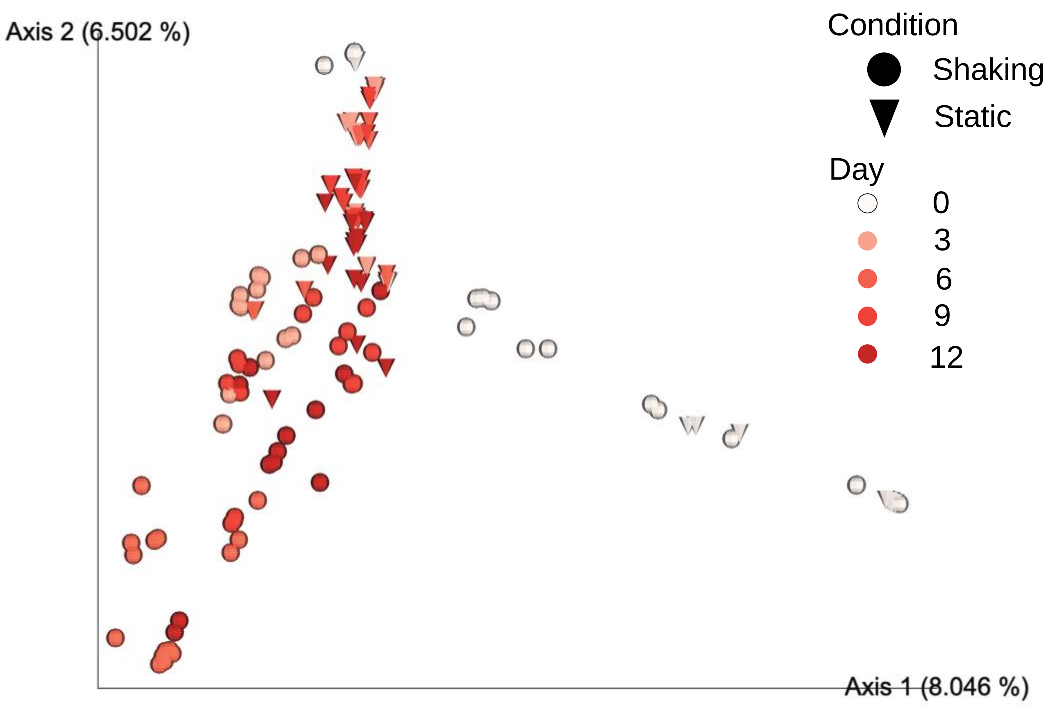

Supplemental Figure 3: Principal Coordinate Analysis of metabolomes including Day 0 samples using Bray-Curtis distances. Shape refers to cultivation condition, color refers to time. Figure was created using GNPS and QIIME2 EMPeror plotting (Bolyen et al., 2019; Vázquez-Baeza et al., 2013)

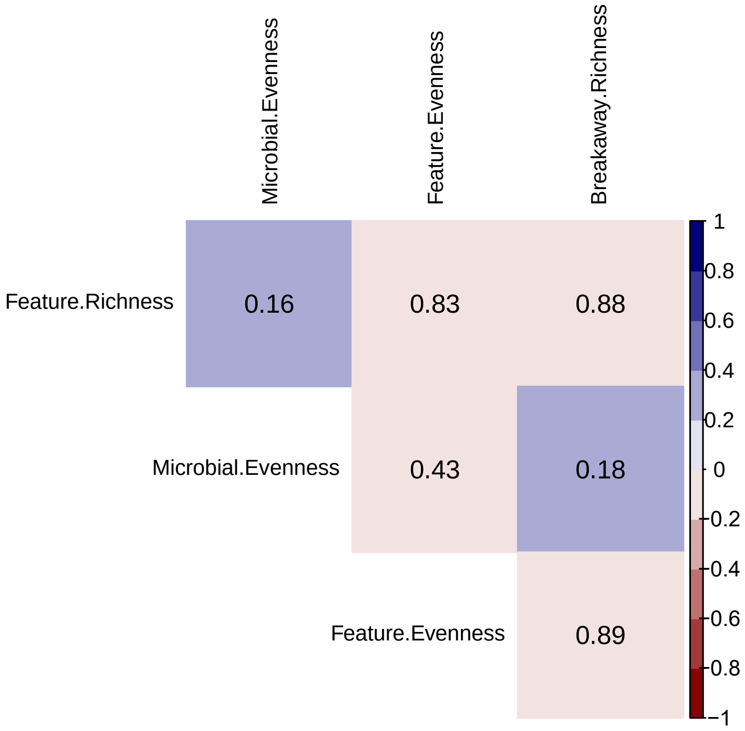

Supplemental Figure 4: Spearman's Rank correlation between alpha diversity metrics. Blue corresponds to positive correlations; red corresponds to negative correlations. P-values are shown in figure for each combination.

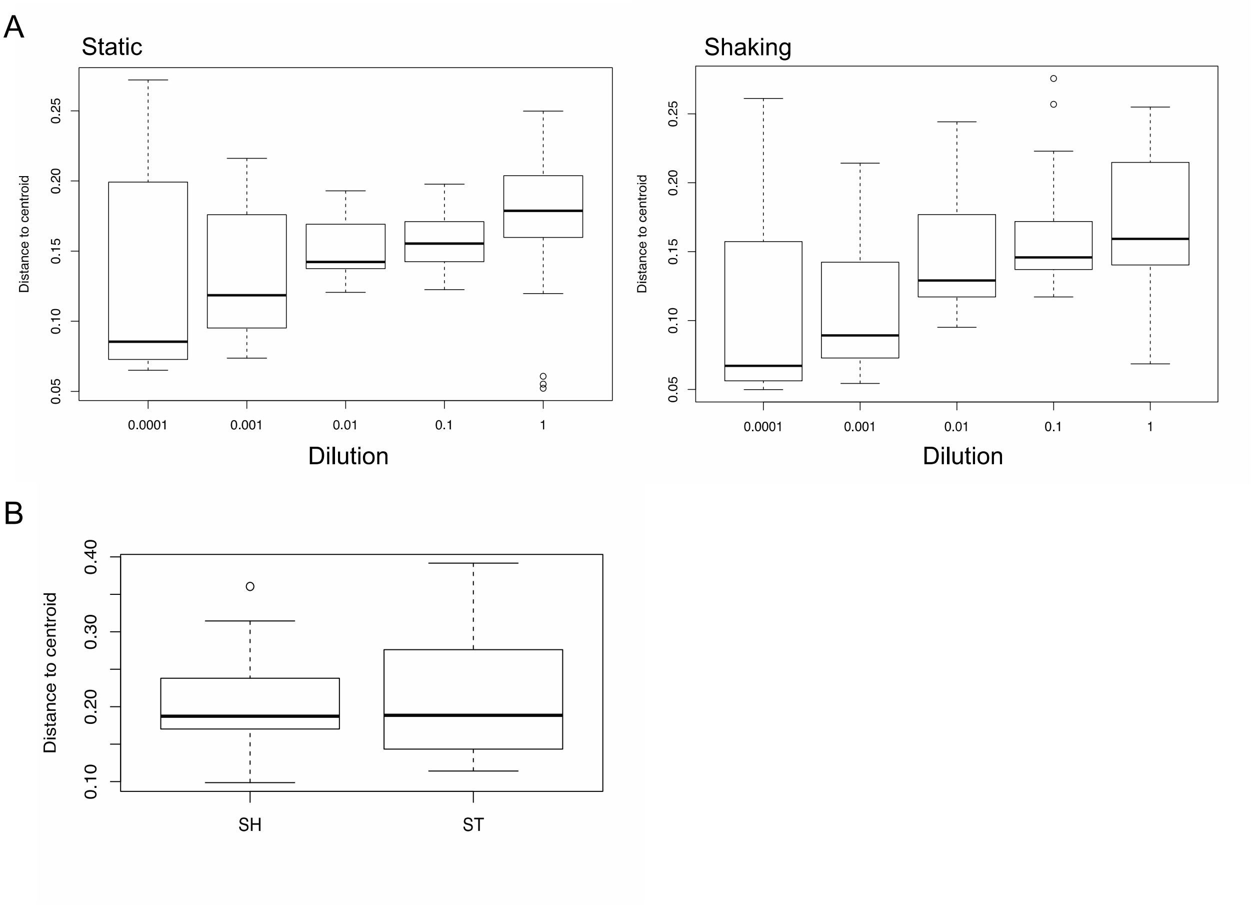

Supplemental Figure 5: Boxplots showing dispersion of microbial communities by (A) dilutions or (B) condition. Large ranges in boxplots indicated high variability between samples. SH refers to shaking, ST refers to static.

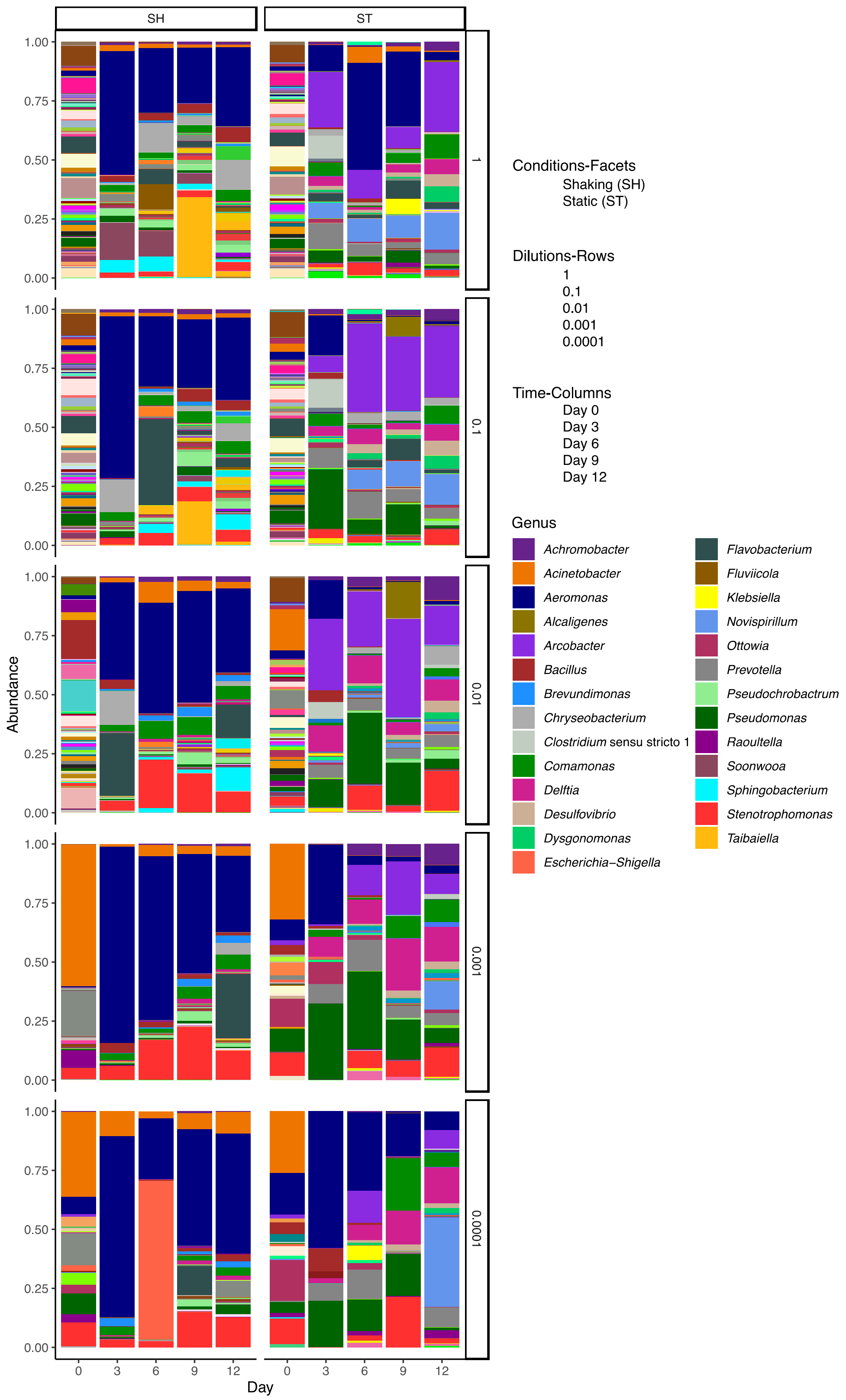

Supplemental Figure 6: Taxonomic bar charts at genus level.

2-heptylquinolin-4(1H)-one (HHQ)

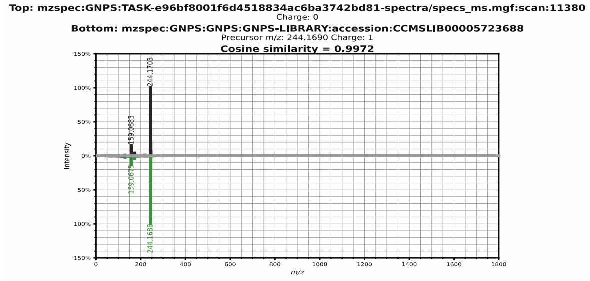

NQNO

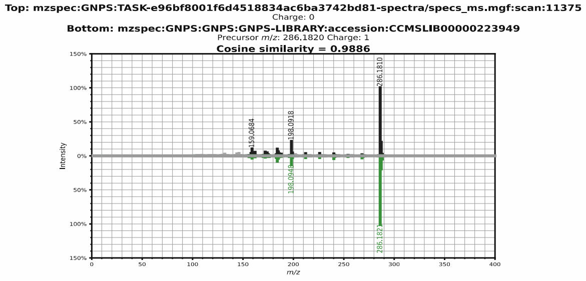

2-nonylquinolin-4(1H)-one (NHQ)

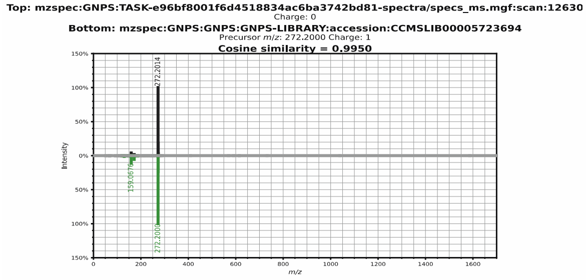

4-hydroxy-2-octylquinoline 1-oxide (HAQ)

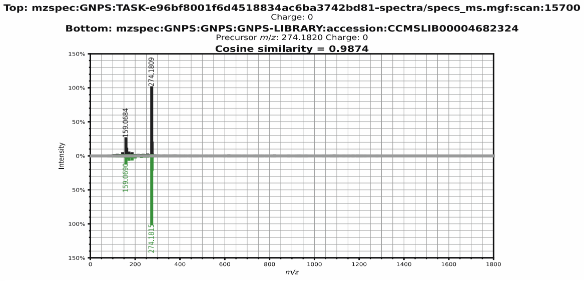

Pseudomonas quinolone signal

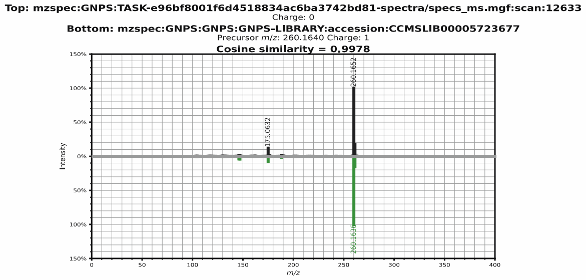

Anisomycin

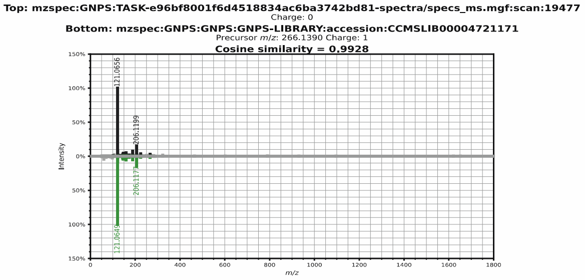

Putative Orfamide A

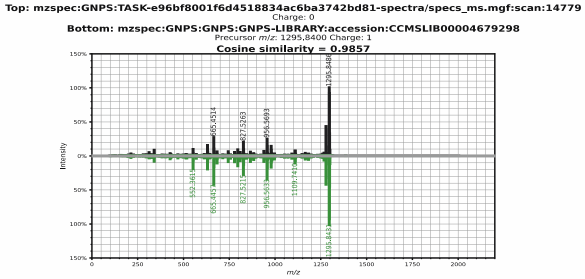

Supplemental Figure 7: Mirror plots for each feature referred to in text. Black is the experimental spectrum while green is the database match.

Anisomycin-19477

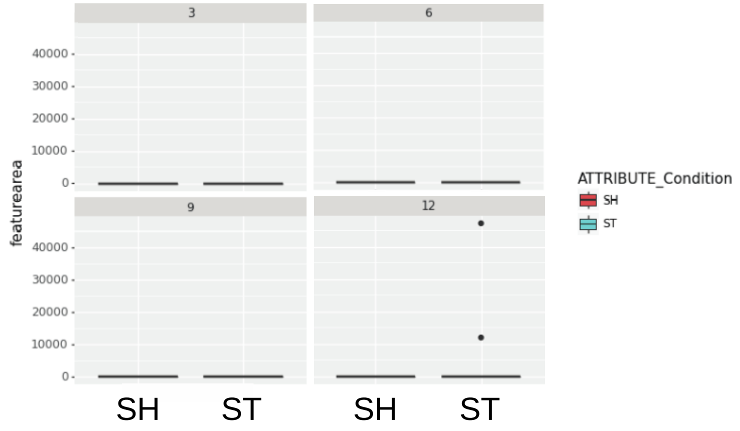

Putative Orfamide A-14779

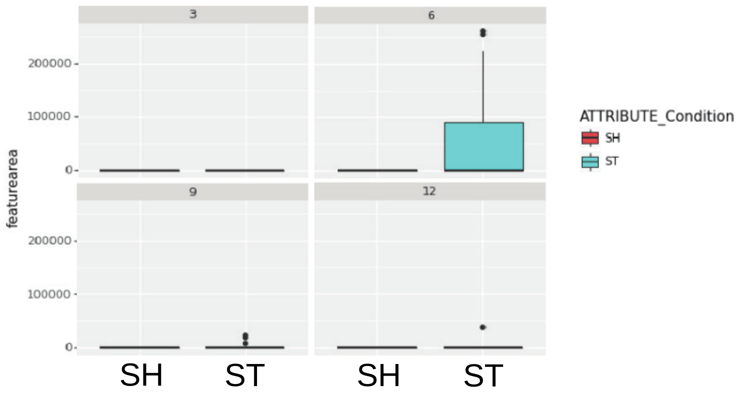

Supplemental Figure 8: Differential abundances for features matched to anisomycin and orfamide A for shaking (SH) and static (ST) conditions. Dilutions are combined for each time point. Figure generated from Feature-Based Molecular Networking (GNPS) (Nothias et al. 2020).

Supplemental References

1. Bartley, L. E., Peck, M. L., Kim, S. R., Ebert, B., Manisseri, C., Chiniquy, D. M., Sykes, R., Gao, L., Rautengarten, C., Vega-Sánchez, M. E., Benke, P. I., Canlas, P. E., Cao, P., Brewer, S., Lin, F., Smith, W. L., Zhang, X., Keasling, J. D., Jentoff, R. E., … Ronald, P. C. (2013). Overexpression of a BAHD acyltransferase, OsAt10, alters rice cell wall hydroxycinnamic acid content and saccharification. *Plant Physiology*, *161*(4), 1615–1633. https://doi.org/10.1104/pp.112.208694
2. Frank E Harrell Jr. (2020). *Hmisc: Harrell Miscellaneous* (R package version 4.3-1). https://cran.r-project.org/package=Hmisc
3. Wei, T., & Simko, V. (2017). *R package "corrplot": Visualization of a Correlation Matrix* (0.84).
4. Bolyen, E., Rideout, J. R., Dillon, M. R., Bokulich, N. A., Abnet, C. C., Al-Ghalith, G. A., Alexander, H., Alm, E. J., Arumugam, M., Asnicar, F., Bai, Y., Bisanz, J. E., Bittinger, K., Brejnrod, A., Brislawn, C. J., Brown, C. T., Callahan, B. J., Caraballo-Rodríguez, A. M., Chase, J., … Caporaso, J. G. (2019). Reproducible, interactive, scalable and extensible microbiome data science using QIIME 2. *Nature Biotechnology*, *37*(8), 852–857. https://doi.org/10.1038/s41587-019-0209-9
5. Vázquez-Baeza, Y., Pirrung, M., Gonzalez, A., & Knight, R. (2013). EMPeror: A tool for visualizing high-throughput microbial community data. *GigaScience*, *2*(1), 2–5. https://doi.org/10.1186/2047-217X-2-16
